# Downregulation of LINC00665 suppresses the progression of lung adenocarcinoma via regulating miR-181c-5p/ZIC2 axis

**DOI:** 10.18632/aging.203240

**Published:** 2021-07-07

**Authors:** Wei Wei, Xiaoliang Zhao, Jiang Liu, Zhenfa Zhang

**Affiliations:** 1 Department of Lung Cancer, Tianjin Medical University Cancer Institute and Hospital, National Clinical Research Center for Cancer, Key Laboratory of Cancer Prevention and Therapy, Tianjin’s Clinical Research Center for Cancer, Tianjin Lung Cancer Center, Tianjin 300060, China; 2 Department of Molecule Imaging and Nuclear Medicine in Diagnosis and Treatment, Tianjin Medical University Cancer Institute and Hospital, National Clinical Research Center for Cancer, Key Laboratory of Cancer Prevention and Therapy, Tianjin’s Clinical Research Center for Cancer, Tianjin Lung Cancer Center, Tianjin 300060, China

**Keywords:** LINC00665, miR-181c-5p, ZIC2, HOXA1, HOXA11

## Abstract

Long non-coding RNA (lncRNA) LINC00665 was demonstrated to be upregulated in lung adenocarcinoma (LUAD) and target miR-181c-5p. ZIC2, which is upregulated in LUAD, serves as a putative target of miR-181c-5p. In this study, we aimed to reveal whether LINC00665 regulates miR-181c-5p/ZIC2 axis to promote LUAD progression. The results showed that LINC00665, HOXA1, ZIC2, and HOXA11 levels were increased in LUAD tissues, while miR-181c-5p level was decreased when compared to the adjacent normal tissues. High expression levels of LINC00665, ZIC2, HOXA1 and HOXA11, and low expression of miR-181c-5p were closely linked to poor prognosis of LUAD patients. Knockdown of LINC00665 induced obvious inhibitions in cell viability, clone formation, invasion and tumorigenesis in LUAD cells, whereas miR-181c-5p downregulation significantly neutralized these effects. In addition, downregulation of ZIC2 obviously reversed the enhancements of cell viability, clone formation, invasion and tumorigenesis induced by miR-181c-5p knockdown. In summary, the present study reveals that silencing of LINC00665 suppresses LUAD progression through targeting miR-181c-5p/ZIC2 axis.

## INTRODUCTION

Lung cancer is the most common malignancy and accounts for the leading cause of cancer-related death all over the world, based on the Cancer Statistics published by American Cancer Society in 2018 [1]. Lung adenocarcinoma (LUAD) as the most common type of lung cancer, has a 5-year survival rate of only 15% [2]. LUAD is characterized by duct formation and mucus generation, and can be classified mainly into acinar, lepidic, papillary, solid and micropapillary histological subtypes.

Long noncoding RNAs (lncRNAs) are a new class of non-protein-coding RNAs with length from 200 nt to ~100 kilobases [3, 4]. Accumulated evidence has illustrated that lncRNAs play important roles in multiple kinds of cellular processes, such as cell growth, apoptosis, differentiation, invasion and migration [5–8]. Importantly, lncRNAs have been identified to be frequently deregulated in cancers and function as oncogenes or tumor suppressors [9, 10]. Long intergenic non-coding RNA 00665 (LINC00665) has been reported to serve as an oncogene in several kinds of cancers, including gastric cancer [11, 12], breast cancer [13], prostate cancer [14] and colorectal cancer [15]. Liu et al. [16] found that LINC00665 expression was increased in lung cancer tissues and cells with acquired gefitinib resistance, and downregulation of it restored gefitinib sensitivity, indicating that LINC00665 may be involved in lung cancer progression. Bioinformatics analysis showed a high expression pattern of LINC00665 in LUAD, but its role and the underlying mechanism in LUAD progression still remain unknown.

MicroRNAs (miRNAs, about 18-25 nt) are another common type of noncoding RNAs which post-transcriptionally modulate gene expression via base-paring to the target mRNAs [17]. Noticeably, lncRNA could serve as competing endogenous RNA (ceRNA) to sponge miRNAs, promoting the transcription of miRNA targets [18]. Zic family member 2 (ZIC2) has been reported to be overexpressed in LUAD and promotes cancer stemness [19]. In this study, we applied the starBase online software to predict the target of LINC00665 and found that LINC00665 might target miR-181c-5p/ZIC2 axis.

In the current study, we aimed to investigate the effect of LINC00665 on the development and progression of LUAD, and to uncover whether miR-181c/5p/ZIC2 axis was involved.

## RESULTS

### Bioinformatics analysis of LINC00665 expressions and clinical values in LUAD

**Figure 1:**
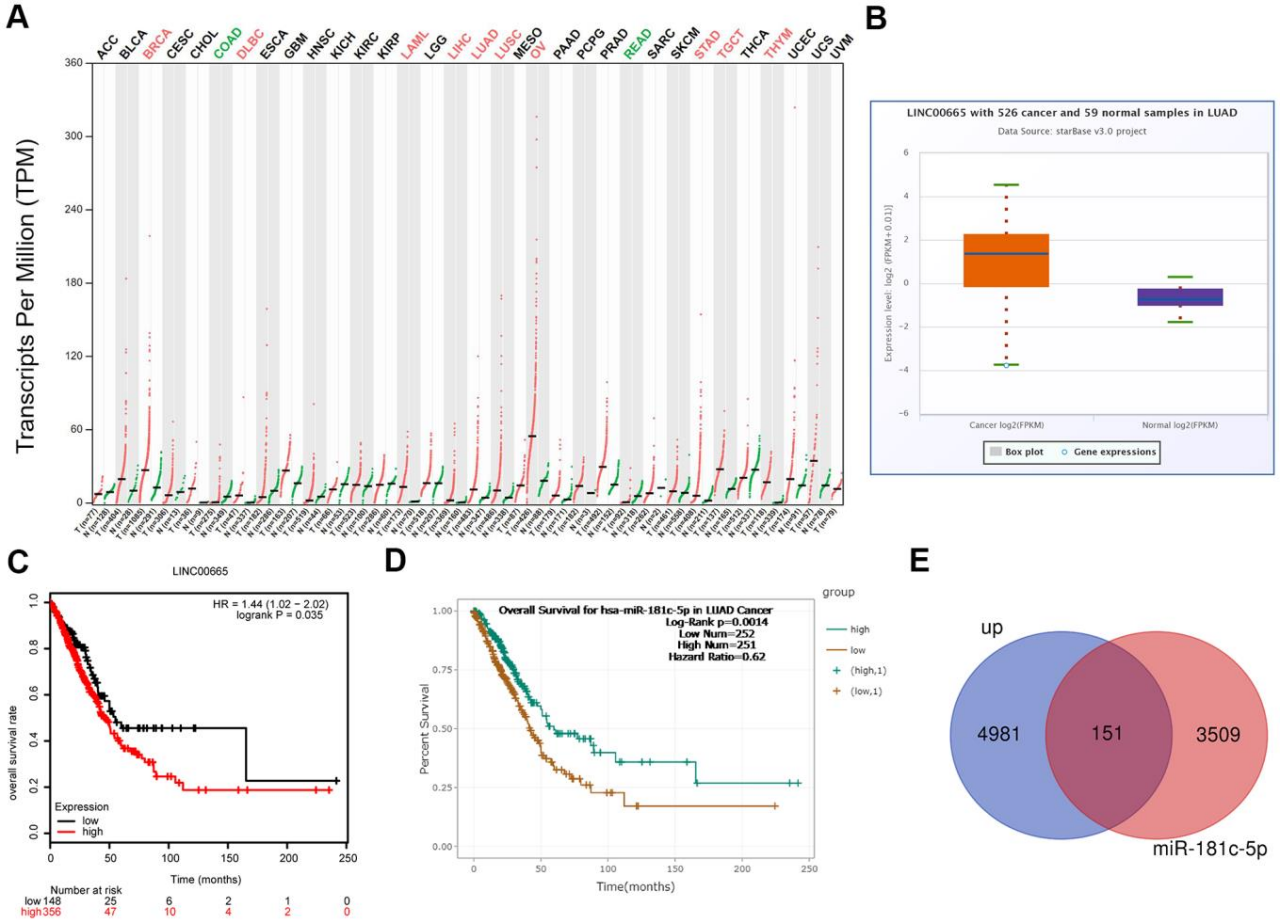
**Bioinformatics analysis of the expression and clinical value of LINC00665 in LUAD.** (**A**) The expression patterns of LINC00665 in different kinds of cancers were identified using GEPIA software. (**B**) StarBase bioinformatics method was applied to analyze the different expression levels of LINC00665 in LUAD tissues and the normal samples. (**C**) TCGA database showed that high expression of LINC00665 predicted a poor prognosis in LUAD patients. (**D**) StarBase bioinformatics method was used to assess the relationship between miR-181c-5p expression levels of the overall survival in LUAD. (**E**) Venn image showing the genes which was not only upregulated in LUAD but also under the regulation of miR-181c-5p.

To reveal the function of LINC0065 in the progression of LUAD, we first assessed its expression patterns and clinical values in LUAD using bioinformatics methods. The results from GEPIA and starBase databases demonstrated that LINC00665 showed a higher expression pattern in LUAD samples as compared to normal samples (Figure 1A, 1B), and the high expression of LINC00665 predicted lower overall survival rate (Figure 1C). In addition, we evaluated the clinical value of miR-181c-5p, the target gene of LINC00665, via bioinformatics methods. The results showed that patients with miR-181c-5p low expression had shorter overall survival time than patients with miR-181c-5p high expression (Figure 1D). Moreover, we applied GO analysis to assess the enriched functions of genes which were upregulated in LUAD and also targeted by miR-181c-5p. A total of 151 upregulated genes in LUAD were identified to serve as the predicted target genes of miR-181c-5p (Figure 1E). These151 genes were enriched in nucleus of the cellular component term (Supplementary Figure 1A, 1B). These above findings suggested LINC00665 and miR-181c-5p might be involved in the progression of LUAD.

### Bioinformatics analysis of the expressions of ZIC2, HOXA1 and HOXA11 in LUAD

**Figure 2:**
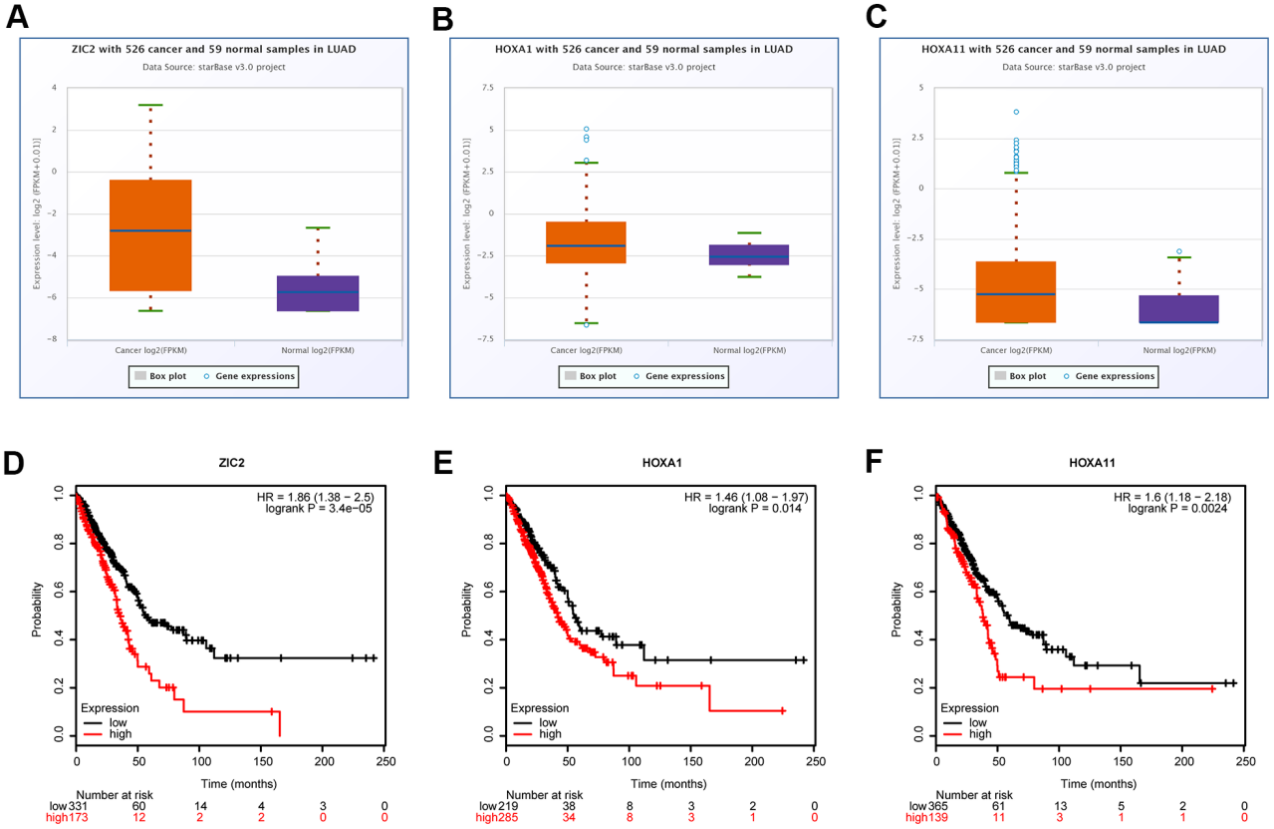
**Bioinformatics analysis of the expression correlations and clinical values of ZIC2, HOXA1 and HOXA11 in LUAD.** (**A**–**C**) StarBase bioinformatics method was used for analyze the levels of ZIC2, HOXA1 and HOXA11 in LUAD tissues and the normal samples. (**D**–**F**) TCGA database was used to evaluate the relationships between the expression levels of ZIC2, HOXA1 and HOXA11 and the overall survival rates in LUAD patients.

Evidence has demonstrated that homeobox (HOX) A1 (HOXA1) and HOXA11 play oncogenic roles in lung cancer [20, 21], and they are the putative target genes of miR-181c-3p, as same as ZIC2, as analyzed by the bioinformatics analysis using the starBase database. Thus, we also assessed the expression patterns of HOXA1 and HOXA11 in LUAD using the bioinformatics methods. Compared with the normal group, the expression levels of ZIC2, HOXA1 and HOXA11 were significantly elevated in LUAD tissues (Figure 2A, 2C). Noticeably, miR-181c-5p expression was negatively correlated with the expression levels of LINC00665 (Supplementary Figure 2A), ZIC2 (Supplementary Figure 2B), HOXA1 (Supplementary Figure 2C) and HOXA11 (Supplementary Figure 2D). In addition, ZIC2 expression showed a positive correlation with the expression levels of HOXA1 (Supplementary Figure 2E) and HOXA11 (Supplementary Figure 2F). And, the high expression levels of ZIC2, HOXA1 and HOXA11 were strongly related to poor prognosis of LUAD patients (Figure 2D–2F). These results demonstrated that ZIC2 was overexpressed in LUAD and linked to poor prognosis.

### Evaluation of the crosstalk between LINC00665, miR-181c-5p and ZIC2 in LUAD

**Figure 3:**
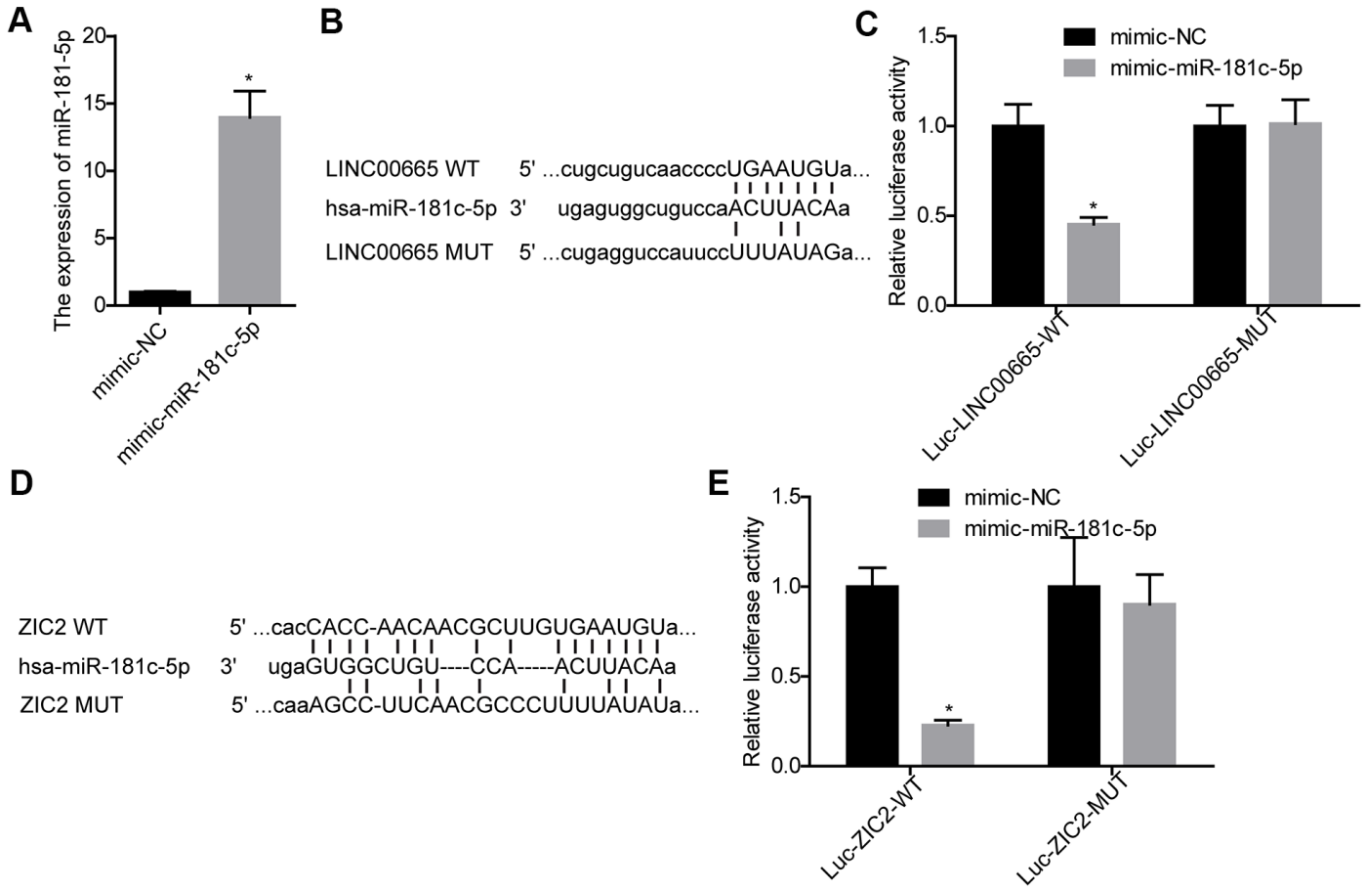
**Evaluation of the relationship between LINC00665 and miR-181c-5p, and miR-181c-5p and ZIC2.** (**A**) The expression of miR-181c-5p was detected by RT-PCR after SK-LU-1 cells were transfected with mimic-miR-181c-5p or mimic-NC. (**B**) Predicted binding sites between LINC00665 and miR-181c-5p. (**C**) Luciferase gene reporter assay was carried out to verify whether miR-181c-5p was a target gene of LINC00665 in SK-LU-1 cells. (**D**) A schematic drawing indicated the putative binding sites between miR-181c-5p and ZIC2. (**E**) Luciferase gene reporter assay was carried out to verify whether ZIC2 was a target gene of miR-181c-5p in SK-LU-1 cells. (^*^p<0.05).

Next, we carried out luciferase gene reporter assay to verify the relationship between LINC00665 and miR-181c-5p, and miR-181c-5p and ZIC2. The expression of miR-181c-5p was significantly increased when SK-LU-1 cells were infected with mimic-miR-181c-5p (Figure 3A). We then constructed the WT and MUT luciferase reporter vectors of LINC00665, and the sequences were shown in Figure 3B. The luciferase activity was decreased when miR-181c-5p was upregulated in SK-LU-1 cells, whereas mutations of the binding sites abolished this effect (Figure 3C). Also, miR-181c-5p upregulation significantly reduced the luciferase activity of luc-ZIC2-WT vector, and mutations of the binding sites abolished it (Figure 3D, 3E). These results demonstrated that LINC00665 could target miR-181c-5p/ZIC2 axis.

### Evaluation of the expression levels and clinical values of LINC00665, miR-181c-5p, ZIC2, HOXA1 and HOXA11 in LUAD

**Figure 4:**
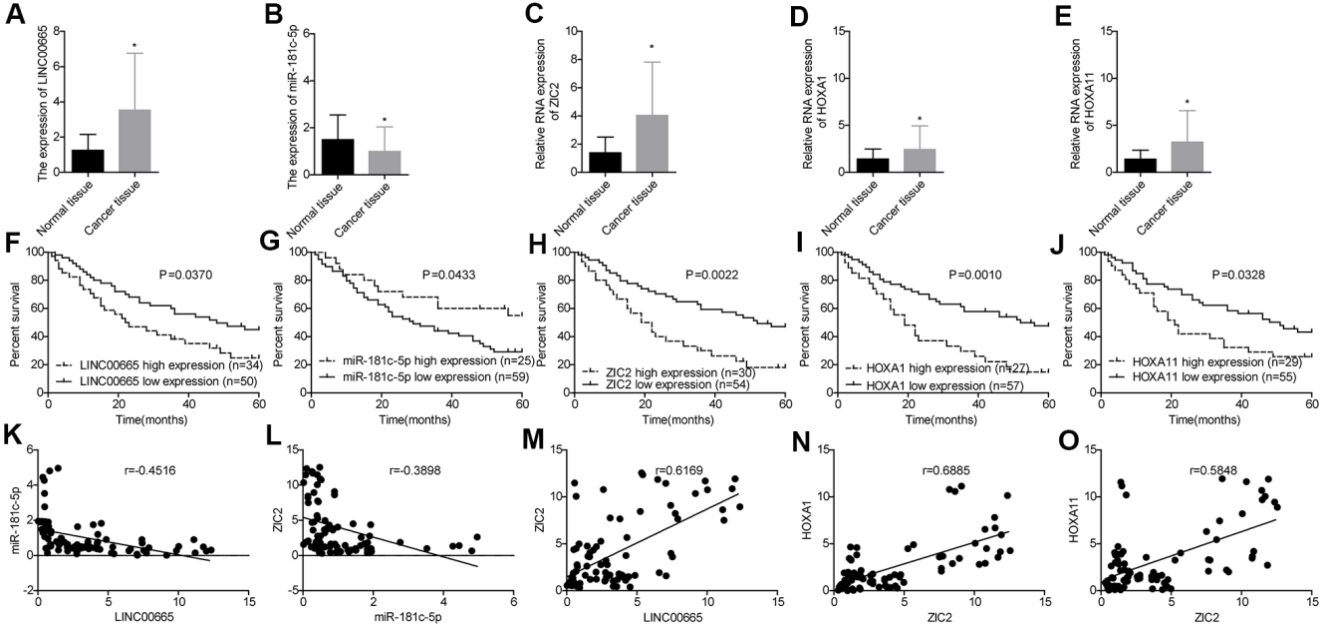
**Evaluation of the expression levels of LINC00665, miR-181c-5p, ZIC2, HOXA1 and HOXA11 in LUAD tissues and their clinical values.** (**A**–**E**) The expression of LINC00665, miR-181c-5p, ZIC2, HOXA1 and HOXA11 in 84 paired LUAD tissues and the paracancerous normal tissues were tested by RT-PCR technology. (**F**–**J**) Overall survival analysis of the effects of different expression levels of LINC00665, miR-181c-5p, ZIC2, HOXA1 and HOXA11 in the prognosis of LUAD patients. (**K**–**O**) Pearson correlation analysis of the correlations between LINC00665, ZIC2, miR-181c-5p, HOXA1 and HOXA11. (^*^p<0.05).

Next, we used RT-PCR assay to further assess the expression profiles of LINC00665, miR-181c-5p, ZIC2, HOXA1 and HOXA11 in 84 paired LUAD tissues and the paracancerous normal tissues. LINC00665 (Figure 4A), ZIC2 (Figure 4C), HOXA1 (Figure 4D) and HOXA11 (Figure 4E) levels were elevated in LUAD tissues, while miR-181c-5p expression was decreased (Figure 4B). High expression levels of LINC00665, ZIC2, HOXA1 and HOXA11 and low expression level of miR-181c-5p were closely associated with the shorter overall survivals in LUAD patients (Figure 4F–4J). Moreover, Pearson correlation analysis showed that miR-181c-5p expression was negatively correlated with LINC00665 and ZIC2 expression levels in LUAD tissues (Figure 4K, 4L), and ZIC2 expression level showed positive correlations with LINC00665, HOXA1 and HOXA11 expressions (Figure 4M–4O). These results confirmed that LINC00665, ZIC2, HOXA1 and HOXA11 were overexpressed, and miR-181c-5p was downregulated in LUAD.

### Knockdown of LINC00665 inhibits cell growth and invasion and reduces ZIC2 expression via targeting miR-181c-5p in LUAD cells

**Figure 5:**
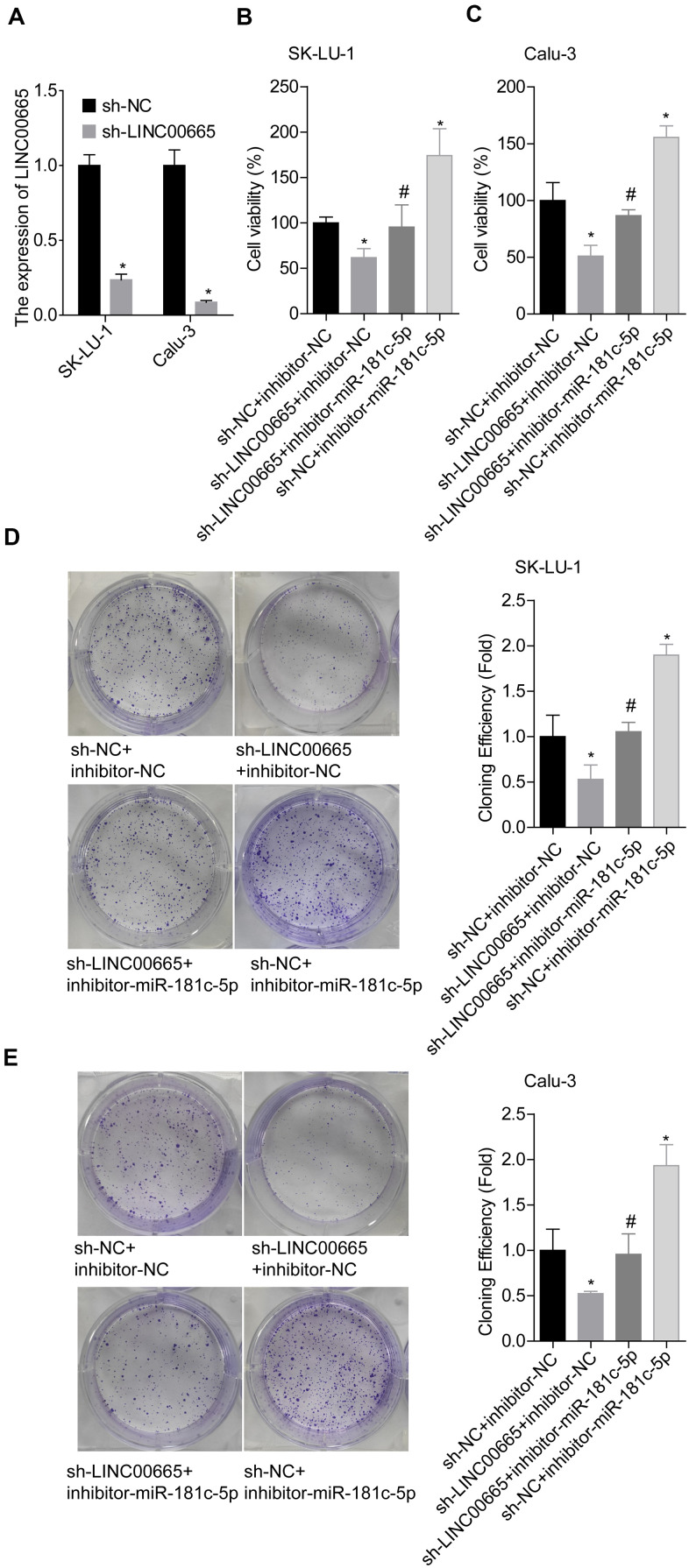
**Assessment of the function of LINC00665/miR-181c-5p axis in modulating LUAD cell growth.** (**A**) The expression of LINC00665 was detected by RT-PCR after sh-LINC00665 was stably transfected into SK-LU-1 and Calu-3 cells. Stably transfected SK-LU-1 and Calu-3 cells (sh-NC + inhibitor-NC, sh-LINC00665 + inhibitor-NC, sh-LINC00665 + inhibitor-miR-181c-5p, sh-NC + inhibitor-miR-181c-5p) were collected and subjected to the following assays. (**B**, **C**) Cell viability was assessed by CCK-8 assay. (**D**, **E**) Cell clone formation was detected by clone formation assay. (^*^p<0.05, vs. sh-NC + inhibitor-NC group; ^#^*p<*0.05, vs. sh-LINC00665 + inhibitor-NC group).

**Figure 6:**
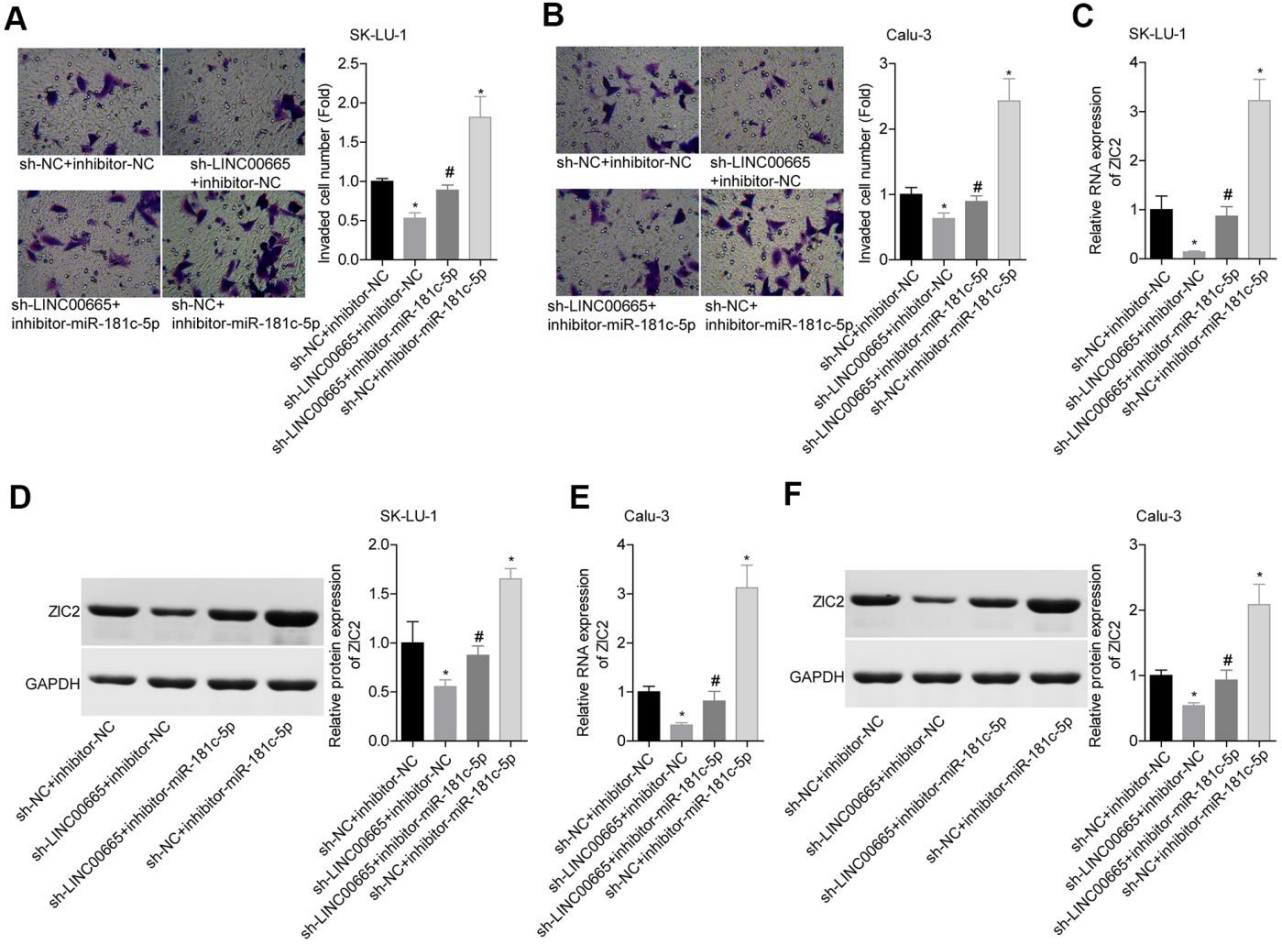
**Assessment of the function of LINC00665/miR-181c-5p axis in regulating LUAD cell invasion and ZIC2 expression.** (**A**, **B**) Transwell chambers were applied for cell invasion assessment in sh-NC + inhibitor-NC, sh-LINC00665 + inhibitor-NC, sh-LINC00665 + inhibitor-miR-181c-5p, sh-NC + inhibitor-miR-181c-5p groups. (**C**, **E**) The mRNA level of ZIC2 was detected by RT-PCR technology. (**D**, **F**) The protein level of ZIC2 was measured by western blotting technology. (^*^p<0.05, vs. sh-NC + inhibitor-NC group; ^#^*p<*0.05, vs. sh-LINC00665 + inhibitor-NC group).

We then elucidated the effects of LINC00665/miR-181c-5p on the progression of LUAD *in vitro*. The expression of LINC00665 was apparently reduced when sh-LINC00665 was stably introduced to SK-LU-1 and Calu-3 cells (Figure 5A). Cell viability (Figure 5B, 5C), clone formation (Figure 5D, 5E) and invasion (Figure 6A, 6B) abilities were significantly weakened when -LINC00665 was downregulated in SK-LU-1 and Calu-3 cells, while inhibitor-miR-181c-5p caused the opposite results and reversed the above tendencies induced by sh-LINC00625 (Figures 5B–5E, 6A, 6B). In addition, knockdown of LINC0000665 significantly reduced ZIC2 expression (Figure 6C–6F), whereas this potency was abolished when miR-181c-5p was downregulated in SK-LU-1 and Calu-3 cells. These findings suggested that downregulation of LINC00665 repressed cell growth and invasion, and reduced ZIC2 expression in LUAD through targeting miR-181c-5p.

### Knockdown of miR-181c-5p promotes LUAD cell growth and invasion via directly regulating ZIC2 expression

**Figure 7:**
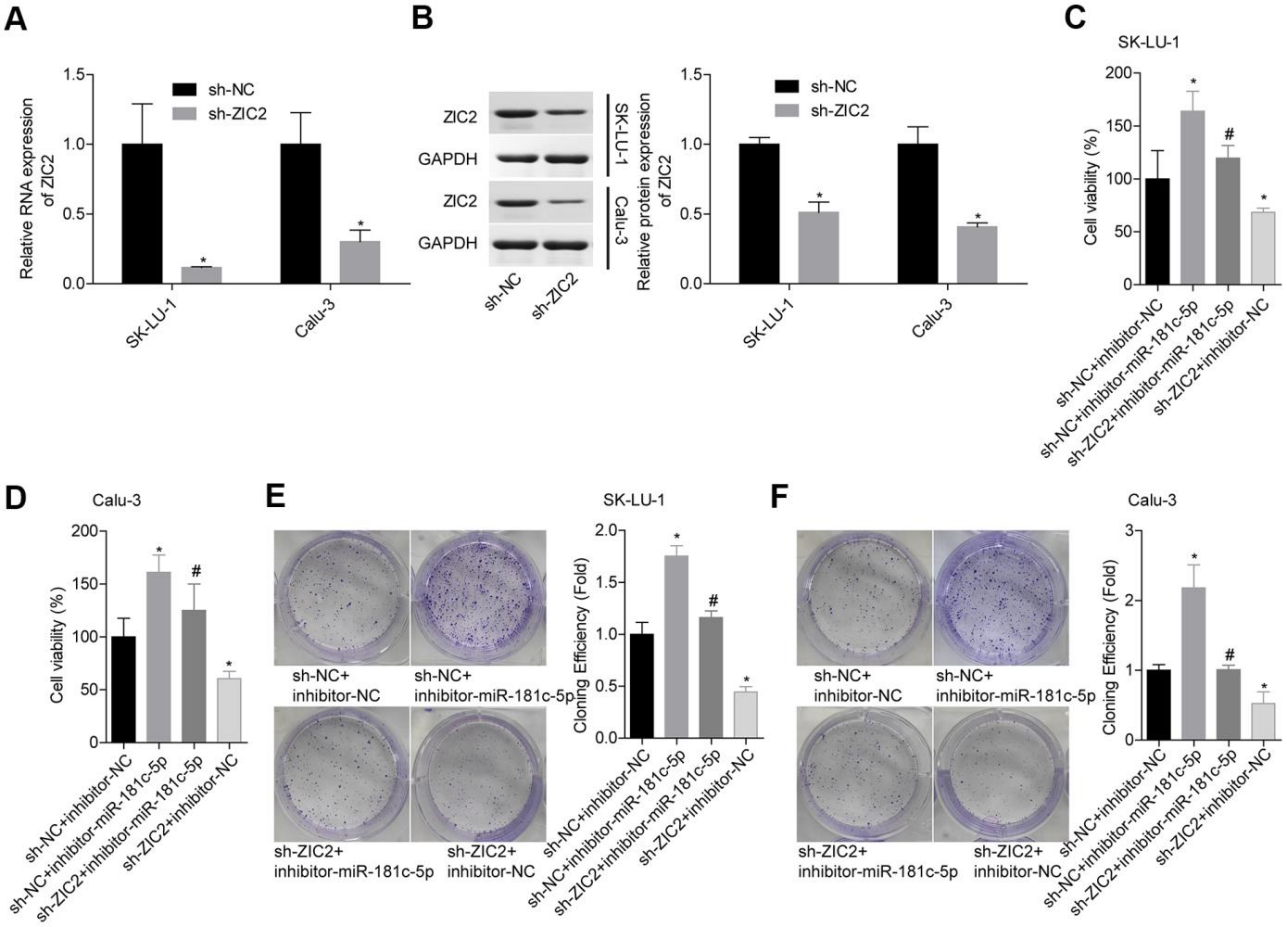
**Knockdown of miR-181c-5p inhibited LUAD cell growth via targeting ZIC2.** (**A**, **B**) The knockdown efficiencies of sh-ZIC2 at mRNA and protein levels were detected by RT-PCR and western blotting technologies. Then, SK-LU-1 and Caki-1 cells with sh-NC + inhibitor-NC, sh-NC + inhibitor-miR-181c-5p, sh-ZIC2 + inhibitor-miR-181c-5p or sh-ZIC2 + inhibitor-NC stable transfection were harvested and submitted to the following experiments. (**C**, **D**) Cell viability was assessed by CCK-8 assay. (**E**, **F**) Cell clone formation was detected by clone formation assay. (^*^p<0.05, vs. sh-NC + inhibitor-NC group; ^#^*p<*0.05, vs. sh-NC + inhibitor-miR-181c-5p group).

**Figure 8:**
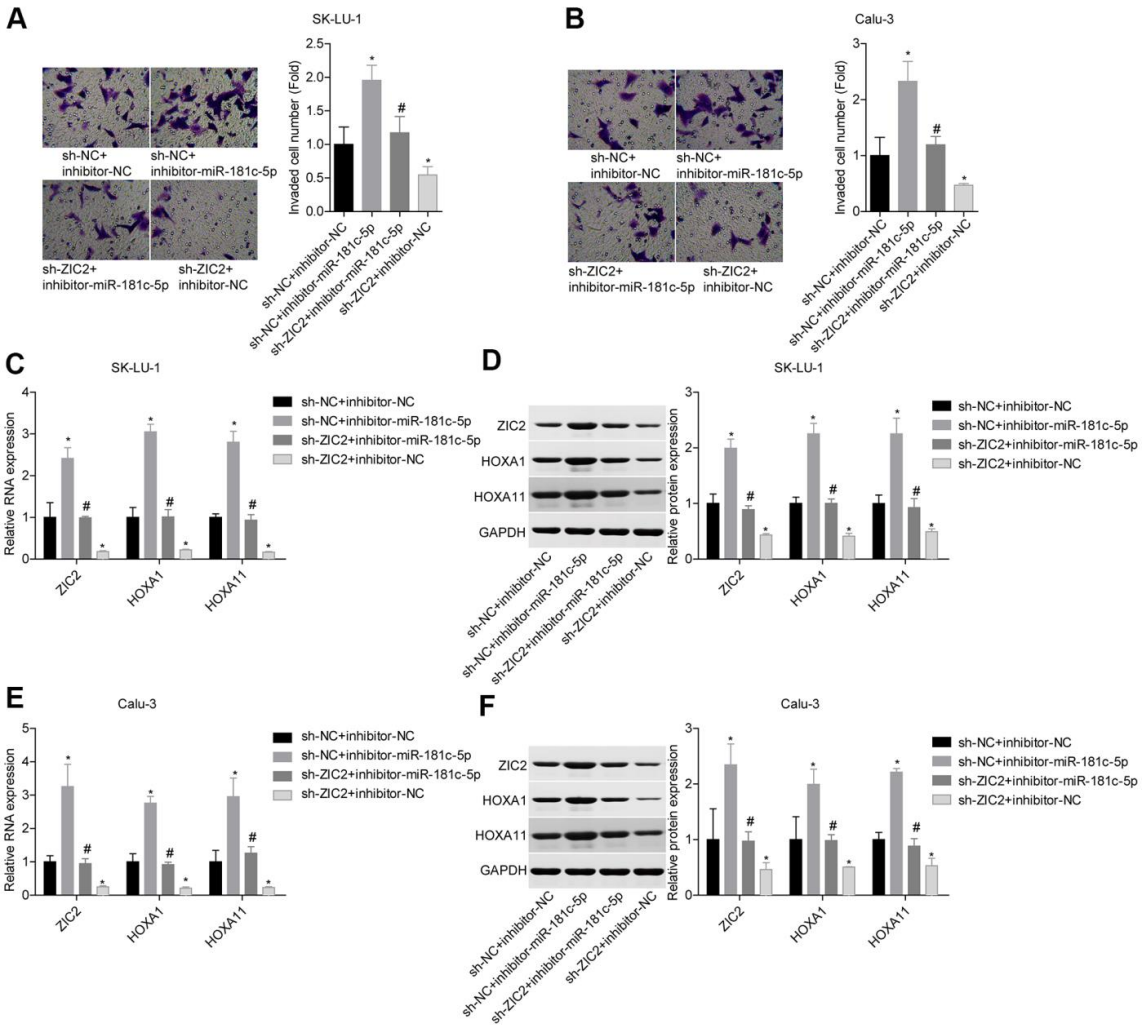
**Knockdown of miR-181c-5p inhibited LUAD cell invasion by targeting ZIC2.** (**A**, **B**) Transwell chambers were applied for cell invasion assessment. (**C**, **E**) The mRNA levels of ZIC2, HOXA1 and HOXA11 were detected by RT-PCR technology. (**D**, **F**) The protein levels of ZIC2, HOXA1 and HOXA11 were measured by western blotting technology. (^*^p<0.05, vs. sh-NC + inhibitor-NC group; ^#^*p<*0.05, vs. sh-NC + inhibitor-miR-181c-5p group).

Next, we assessed miR-181c-5p/ZIC2 role in LUAD progression *in vitro*. The expression of ZIC2 was apparently decreased when SK-LU-1 and Calu-3 cells were stably transfected with sh-ZIC2 at mRNA (Figure 7A) and protein (Figure 7B) levels. Inhibitor-miR-181c-5p stable transfection significantly enhanced cell viability (Figure 7C, 7D), clone formation (Figure 7E, 7F) and invasion (Figure 8A, 8B), whereas ZIC2 downregulation obviously rescued these tendencies and induced opposite results (Figures 7A–7D, 8A, 8B). In addition, downregulation of miR-181c-5p induced significant increases in the expressions of HOXA1 and HOXA11, whereas this effect was abrogated when ZIC2 was silenced in both SK-LU-1 and Calu-3 cell lines (Figure 8C–8F). These results indicated that miR-181c-5p downregulation promoted LUAD cell growth and invasion by regulating ZIC2 expression.

### Knockdown of LINC00665 suppresses *in vivo* tumor formation via targeting miR-181c-5p/ZIC2 in LUAD

**Figure 9:**
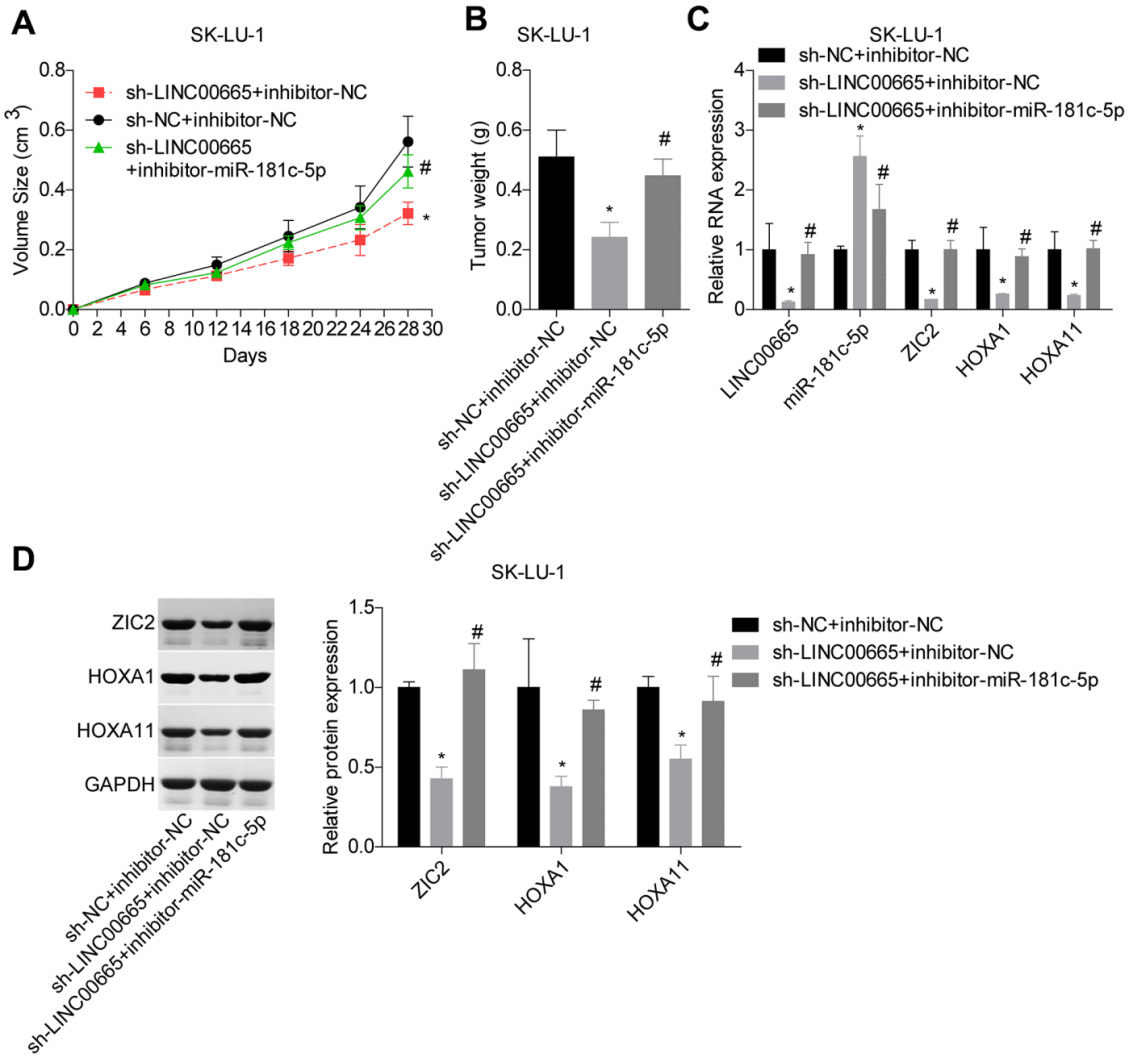
**LINC00665 downregulation repressed tumor growth via regulating miR-181c-5p/ZIC2.** (**A**, **B**) Tumor-bearing mice were constructed to assess the tumorigenesis of SK-LU-1 cells with sh-NC+inhibitor-NC, sh-LINC00665+inhibitor-NC or sh-LINC00665+inhibitor-miR-181c-5p stable transfection. (**C**) RT-PCR technology was applied to test the mRNA levels of LINC00665, miR-181c-5p, ZIC2, HOXA1 and HOX11 in tumors. (**D**) The protein levels of ZIC2, HOXA1 and HOXA11 in mouse tumor tissues were determined by western blotting assay. (^*^p<0.05, vs. inhibitor-NC+sh-NC group; ^#^*p<*0.05, vs. inhibitor-miR-181c-5p+sh-NC group).

At the end, we carried out the *in vivo* tumor-burdened experiment to reveal the role of LINC00665/miR-181c-5p/ZIC2 axis in the progression of LUAD. Tumor weights were significantly reduced when LINC00665 was downregulated in SK-LU-1 cells, whereas it was obviously neutralized by inhibitor-miR-181c-5p (Figure 9A, 9B). We then assessed the expressions of ZIC2, HOXA1 and HOXA11 in mice tumors derived from different groups. LINC00665 downregulation obviously reduced the expressions of ZIC2, HOXA1 and HOXA11, whereas inhibitor-miR-181c-5p reversed this effect (Figure 9C, 9D). These results further demonstrated that knockdown of LINC00665 suppressed *in vivo* tumor formation via targeting miR-181c-5p/ZIC2 in LUAD.

## DISCUSSION

Growing evidence has demonstrated that non-coding RNAs are of importance in all the biological processes detected [22, 23], which added a new layer to our understanding of biological processes. In this study, we elucidated that LINC00665 expression was significantly increased in LUAD tissues, and LINC00665 high expression was closely correlated with patients’ poor prognosis. Importantly, we revealed that LINC0065 served as an oncogene in LUAD via targeting miR-181c-5p/ZIC2 axis.

Up to now, lots of lncRNAs have been identified to be deregulated in LUAD and strongly implicated in its development. For instance, Qin et al. [24] identified a high expression pattern of lncRNA MIR31HG in LUAD tissues and cell lines, which was strongly associated with the N classification, M classification, clinical staging, and prognosis. Guan et al. [25] reported that LINC00673-v4 apparently enhanced cell migration, invasiveness and metastasis in LUAD. Li et al. [26] reported that lncRNA MEG3 showed a high expression profile in LUAD tissues and some specific cell lines, which predicted lower survival probabilities in LUAD patients. In addition, entopic expression of MEG3 significantly promoted cell proliferation, invasion and angiogenesis. LINC00665, a novel lncRNA, has been found to be overexpressed in hepatocellular carcinoma (HCC), and patients with LINC00665 high expression had a worse overall survival rate [27]. Recently, LINC00665 role in cancers has been revealed gradually. Studies have found that LINC00665 served as an oncogene in gastric cancer [11, 12], breast cancer [13], prostate cancer [14] and colorectal cancer [15]. Liu et al. [16] reported that LINC00665 was highly expressed in LUAD tissues, which closely associated with lower survival rate, and silencing of LINC00665 conferred cell gefitinib-sensibility. Congruently, this study revealed that LINC00665 also showed a high expression level in LUAD tissues as demonstrated by GEPIA, and confirmed by RT-PCR. LINC665 high expression was closely related to poor prognosis in patients with LUAD. And, downregulation of LINC00665 significantly inhibited LUAD cell growth and invasion, suggesting that LINC00665 functioned as an oncogene in LUAD.

In mechanism, we identified that LINC00665 could target miR-181c-5p. Low expression of miR-181 has been reported to be frequently found in LUAD [28], and miR-181 low expression was closely correlated to the shorter overall survival in patients with non-small-cell lung cancer (NSCLC) [29]. Wu at al. [30] found that miR-181c-5p restored the sensibility of prostate cancer cells to enzalutamide. However, Gao et al. [31] reported that miR-181c-5p expression was significantly increased in pancreatic cancer cells, which induced a dramatic increase in chemoresistance in pancreatic cancer. These results indicated that miR-181c-5p exerted different roles in various cancers. In the current study, we clarified a low expression profile of miR-181c-5p in the LUAD tissues as compared to that in the adjacent normal tissues, and the low expression of miR-181c-5p was linked to poor prognosis. In addition, we elucidated that miR-181c-5p, negatively regulated by LINC00665 functioned as a tumor suppressive gene in LUAD. Moreover, miR-181c-5p downregulation dramatically impaired sh-LINC00665 roles in repressing cell growth, clone formation, invasion and tumorigenesis in LUAD cells, illustrating that downregulation of LINC00665 inhibited LUAD progression through targeting miR-181c-5p.

Furthermore, we identified that ZIC2 was not only overexpressed in LUAD but also served as a target gene of miR-181c-5p. Our results demonstrated that ZIC2 was upregulated in LUAD tissues, and the high expression of ZIC2 predicted shorter overall survival time in patients with LUAD. In addition, ZIC2 expression negatively correlated with miR-181c-5p expression, while positively correlated with LINC00665 expression in LUAD samples. ZIC2 has been demonstrated to be strongly implicated in several kinds of human malignant cancers. For instance, Zhang et al. [32] found that ZIC2, a target of miR-1284, expressed at a high level in breast cancer, and deficiency of ZIC2 induced significant repressions in cell growth, invasion and migration abilities. ZIC2 also overexpressed in oral squamous cell carcinoma [33], HCC [34], nasopharyngeal carcinoma [35] and epithelial ovarian cancer [36], and served as an oncogene in these kinds of cancers. Wei at al. [19] recently found that ZICs was overexpressed in LUAD and promoted cancer stemness. To reveal whether ZIC2 was involved in miR-181c-5p roles in LUAD progression, we then carried out the rescuing experiments. The results showed that the promotions in cell growth, clone formation, invasion and tumorigenesis induced by inhibitor-miR-181c-5pwere reversed when ZIC2 was depleted, indicating that downregulation of miR-181c-5p accelerated LUAD progression by targeting ZIC2.

HOXA1 and HOXA11 have been identified to play oncogenic roles in lung cancer [20, 21]. In this study, we found that both HOXA1 and HOXA11 were upregulated in LUAD tissues compared to the normal tissues, and the expression levels of both HOXA1 and HOXA11 showed positive correlations with ZIC2 in LUAD cases. Also, it has been reported that ZIC2 serves as a cofactor of the Mbd3-NuRD complex, co-occupying enhancer regions genome-wide and functioning together in regulating the chromatin state and gene expression, such as HOX genes [37]. In this study, we found that HOXA1 and HOXA11 expressions were increased in LUAD cells following miR-181c-5p downregulation, while decreased when ZIC2 was downregulated. And, ZIC2 downregulation abolished inhibitor-miR-181c-5p role in promoting HOXA1 and HOXA11 expression, indicating that downregulation of miR-181c-5p increased HOXA1 and HOXA11 expressions by targeting ZIC2. Using the bioinformatics analysis, we found that HOXA1 and HOXA11 are also the putative target genes of miR-181c-3p. However, we didn’t confirm the relationship between miR-181c-3p and HOXA1/HOXA11 in the present study using luciferase gene reporter assay. This is a main limitation of this study.

Collectively, this study reveals that LINC00665 serves as a ceRNA of miR-181c-5p and then restores ZIC2 expression. In summary, we demonstrate that downregulation of LINC00665 inhibits LUAD progression via targeting miR-181c-5p/ZIC2 axis. This study provides a theoretic evidence of serving LINC00665/miR-181c-5p/ZIC2 as a target for clinical treatment of LUAD.

## MATERIALS AND METHODS

### Bioinformatics analysis

The different expression patterns of LINC00665 in different kinds of cancers were evaluated by GEPIA (http://gepia.cancer-pku.cn/detail.php). starBase (http://starbase.sysu.edu.cn/) was applied to analyze the expression profiles of LINC00665, ZIC2, HOXA1 and HOXA11 in LUAD tissues and normal lung tissues, as well as their clinical values in predicting the overall survival in LUAD patients and their expression correlations. TCGA database was used to find genes which were upregulated in LUAD [absolute value of FC (fold change) >2, *p<*0.01].

### Tissues samples

Eighty-four cases of clinical human LUAD tissue samples and the adjacent paired normal lung tissues were obtained from patients with LUAD who received pneumonectomy at Tianjin Medical University Cancer Institute and Hospital prior to chemoradiotherapy. Tumor types were identified according to the WHO classification (4th edition). All samples were stored at -80° C for further study. The experimental protocols involving human samples were executed in accordance with the Helsinki Declaration and were approved by the ethical committee of Tianjin Medical University Cancer Institute and Hospital. Informed consent was obtained from every participator.

### Cell lines and culture conditions

Two human LUAD cell lines SK-LU-1 and Calu-3 were brought from American Type Culture Collection (ATCC, Manassas, VA, USA), and were grown in Eagle's Minimum Essential Medium (No. 30-2003, ATCC) with 10% fetal bovine serum (FBS; Gibco, MA, USA).

### Stable cell line establishment

Short hairpin RNAs (shRNAs) used to silence LINC00665 (sh-LINC00665) and ZIC2 (sh-ZIC2) in LUAD cell lines, as well as the negative control vector (sh-NC) were synthesized by GenePharma (Shanghai, China). Lentivirus vectors applied to downregulate and upregulate miR-181c-5p expression were also purchased from GenePharma, which were called as inhibitor-miR-181c-5p and mimic-miR-181c-5p.

To establish the stable transfection cells, SK-LU-1 and Calu-3 cells were infected with sh-LINC00665, sh-ZIC2, inhibitor-miR-181c-5p, mimic-miR-181c-5p, control vector or two of them with the help of polybrene (7 μg/ml), followed by incubation with 5 μg/ml puromycin or/and 100 μg/ml G418.

### Real-time quantitative polymerase chain reaction (RT-PCR)

Total RNA isolation from lung tissues and cells was carried out with RNAsimple total RNA extract kit (CWBIO, Jiangsu, China) according to the manufacturer’s protocols. Subsequently, a total of 1 μg of RNA sample was subjected to RT-PCR assay using the Qiagen OneStep RT-PCR Kit (Qiagen Benelux BV, Venlo, The Netherlands). TaqMan miRNA assays (Applied Biosystems; Thermo Fisher Scientific, Inc.) were carried out to detect the relative expression of miR-181c-5p based on the instructions. GAPDH and U6 serve as the normalization controls for mRNA and miRNA, respectively.

LINC00665-forward (F): 5’-ATCTCGGGATCCCACATCCT-3’; Reverse (R): 5’- ACAATGCTCACTGGGAACCA-3’.

ZIC2-F: 5’- CCTTCCGGAGTCTTTGAAGC-3’, R: 5’- ACGTGGGCATGGAGATTAGC-3’;

HOXA1-F: 5’-GGTCCAAGCTATGGCTCACA-3’, R: 5’- GGGTACCCACCACTTACGTC-3’;

HOXA11-F: 5’- GGAAGAGGGCTGCAAATCCT-3’, R: 5’- CACCTCAGGGAACAGTCCAC-3’;

GAPDH-F: 5’-CCACTAGGCGCTCACTGTTCTC-3’; R: 5’-CATGGTGGTGAAGACGCCAG-3’.

### Western blotting assay

Total protein from lung tissues and cells was isolated with radioimmunoprecipitation (RIPA) lysis buffer (Beyotime, Jiangsu, China) and quantified using the BCA Protein Quantification Kit (Solarbio, Beijing, China). Then, 20 μg proteins were submitted to electrophoresis on 10% sodium dodecyl sulfatepolyacrylamide gel, and were then transferred into the polyvinylidene fluoride (PVDF) membranes (Millipore, Billerica, MA, USA). After that, the membranes were blocked with 5% non-fat milk and probed with the primary antibodies against ZIC2 (No. ab150404, Abcam, MA, USA), HOXA1 (No. ab230513, Abcam), HOXA11 (No. ab72591, Abcam) or GAPDH (Abcam) at 4° C overnight. Next, the membranes were incubated with the corresponding secondary antibodies for 1 hour at room temperature. The western blots were visualized using the enhanced chemiluminescence reagents (Millipore) and detected on iBright CL1500 Imaging System (Thermo Fisher Scientific, USA), and analyzed by ImageJ software. GAPDH serves as an internal reference.

### Luciferase gene reporter assay

The binding sites of miR-181c-5p in LINC00665 were predicted using starBase (http://starbase.sysu.edu.cn/). TargetScan (http://www.targetscan.org/vert_71/) and miRDB (http://mirdb.org/) were used to predict the binding sites of miR-181c-5p in the 3’UTR of ZIC2. The wild-type of ZIC2/LINC00665 3’-UTR or the mutant type (MUT) with binding sites nonsense mutations were cloned into the pGL3 vector (Promega, Madison, WI, USA), which were named as Luc-ZIC2-WT/Luc-LINC00665-WT and Luc-ZIC2-MUT/Luc-LINC00665-MUT, respectively. For luciferase gene reporter assay, SK-LU-1 and Caki-3 cells were co-transfected with WT or MUT of LINC00665/ZIC2 and mimic-miR-181c-5p or mimic-NC. After 48 hours, the luciferase activity for each group was measured by using a Dual-luciferase assay system (Promega) according to the manufacturer’s instructions.

### Cell count kit-8 (CCK-8) assay

CCK-8 Kits (MedChemExpress, Shanghai, China) were applied to assess cell viability in SK-LU-1 and Caki-3 cells. In brief, the stable cells (2,000 cells/well) were inoculated in 96-well plates and incubated at 37° C with 5% CO_2_ for 48 hours. Then, the cells were incubated with 10 μl CCK-8 reagent for another 4 hours at 37° C. The absorbance at 450 nm was detected on a microplate reader (Bio-Rad, Hercules, CA, USA).

### Clone formation assay

Cells with stable transfection were placed into 6-well plates with 500 cells in each well. Following 14 days of incubation at 37° C, cells were fixed with methanol for 10 min, and then stained with 0.1% crystal violet (Solarbio) for 10 min. The clone numbers were counted under a microscopy.

### Transwell chamber assay

Matrigel-coated transwell chambers (BD Bioscience, San Diego, CA, USA) were applied for cell invasion detection. A total of 1×10^5^ stably transfected cells were placed into the top chamber with FBS-free medium. After incubation at 37° C for 48 hours, cells on the top of the filter were removed by swabs, while cells on the bottom were fixed by 4% paraformaldehyde and then stained with 1% crystal violet. The invaded cells were counted under a microscope.

### *In vivo* tumorigenic assay

BALB/c nude male mice aged 6-8 weeks (SLAC Laboratory Animal Co. Ltd, Shanghai, China) were used in this *in vivo* assay. SK-LU-1 cells (1x10^6^) with stable transfection of sh-NC + inhibitor-NC, sh-LINC00665 + inhibitor-NC and sh-LINC00665 + inhibitor-miR-181c-5p were implanted subcutaneously into the nude mice. Each group has 5 mice. The tumors were monitored for 4 weeks post-transplantation. Animal study was approved by the Animal Experimental Ethics Committee of Tianjin Medical University Cancer Institute and Hospital.

### Statistical analysis

All of the quantitative data are presented as means ± standard deviation (SD). The statistical analysis was performed by Student’s two-tailed t tests for two groups, and one-way ANOVA followed by Tukey's tests for multiple groups. Pearson correlation analysis was used to assess the correlations between the expression levels of different indicators. Kaplan-Meier with Log Rank test was used to analyze the clinical value of genes in predicting patients’ overall survival. *p* value < 0.05 was set as statistically significant.

## Supplementary Material

Supplementary Figures
