# Supplementary material for: Downregulation of LINC00665 suppresses the progression of lung adenocarcinoma via regulating miR-181c-5p/ZIC2 axis
Source: Aging (Albany NY). 2021 Jul 7;13(13):17499–515. doi: 10.18632/aging.203240 (PMC8312465; doi:10.18632/aging.203240)
Supplement: Supplementary Figures [file aging-13-203240-s001.pdf]

SUPPLEMENTARY FIGURES

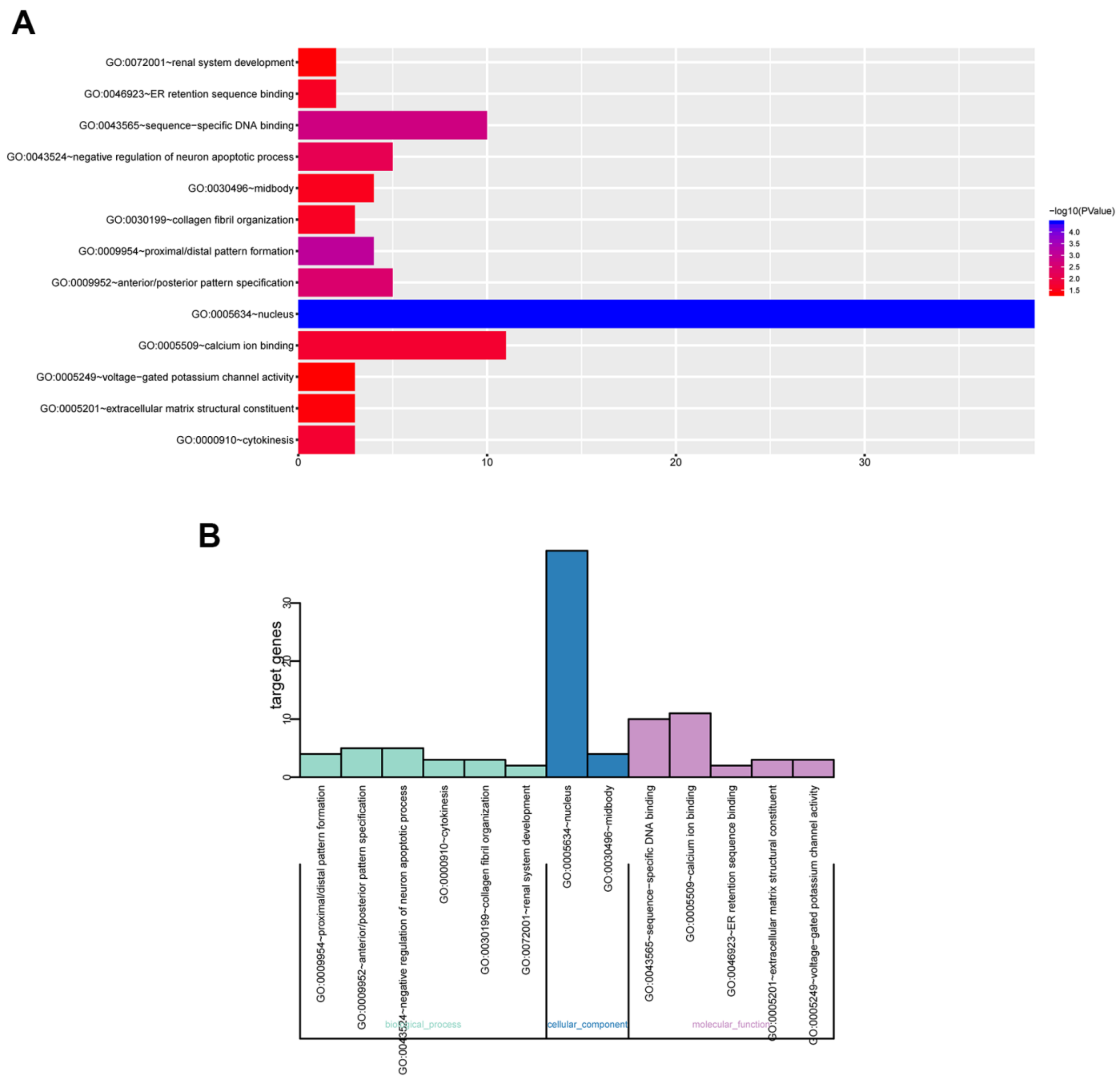

Supplementary Figure 1. GO analysis. (**A**, **B**) GO pathway analysis of the 151 genes which were upregulated in LUAD and also targeted by miR-181c-5p.

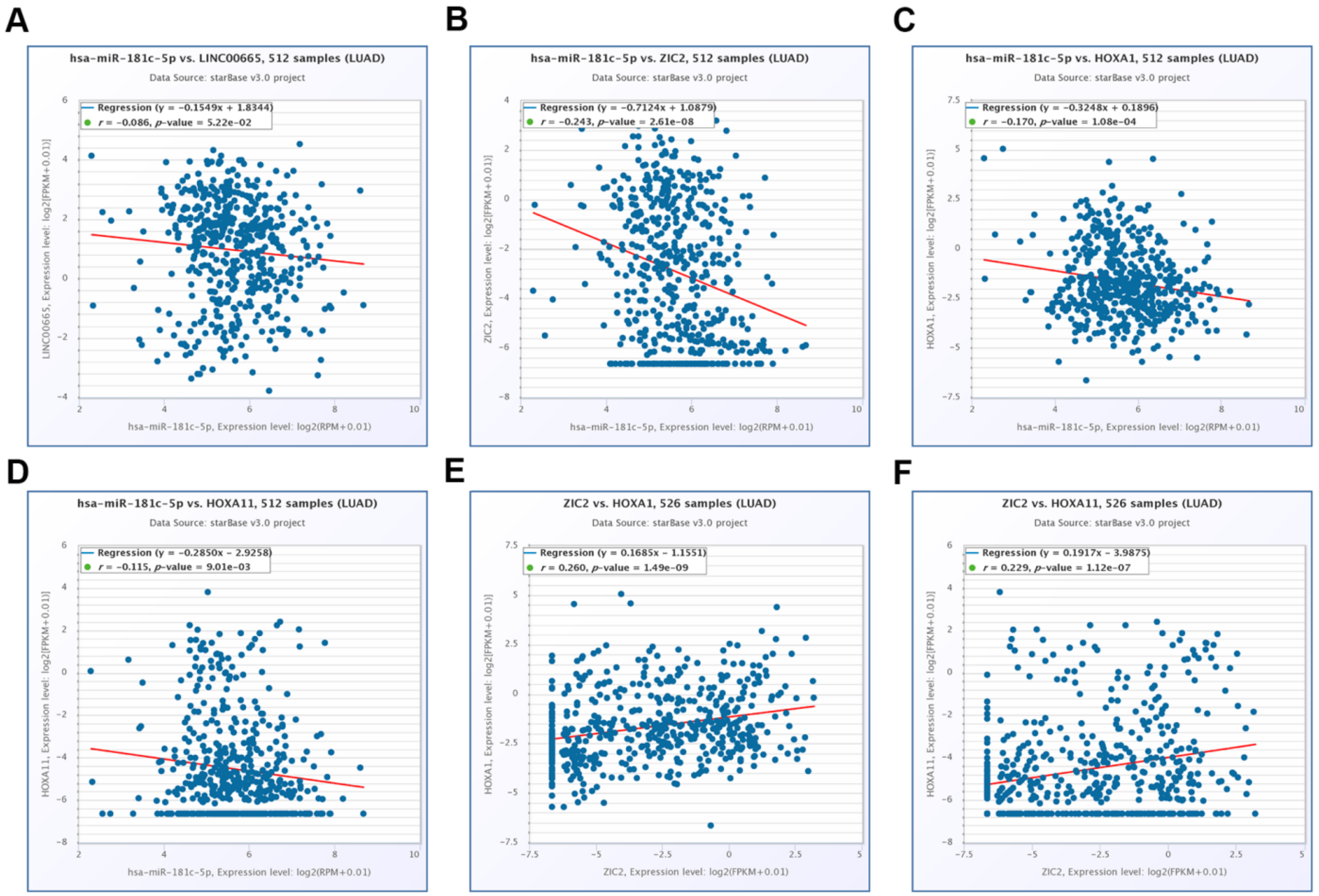

Supplementary Figure 2 Analysis of the correlation of the expression levels of LINC00665, ZIC2, HOXA1, HOXA11 in LUAD samples. (**A**–**F**) StarBase database was applied to analyze the correlations between the expression levels of miR-181c-5p and LINC00665, ZIC2, HOXA1, HOXA11, as well as ZIC2 and HOXA1/HOXA11.
